# Potassium Channel Protein KCNK6 Promotes Breast Cancer Cell Proliferation, Invasion, and Migration

**DOI:** 10.3389/fcell.2021.616784

**Published:** 2021-06-14

**Authors:** Xiangchan Hou, Le Tang, Xiayu Li, Fang Xiong, Yongzhen Mo, Xianjie Jiang, Xiangying Deng, Miao Peng, Pan Wu, Mengyao Zhao, Jiawei Ouyang, Lei Shi, Yi He, Qijia Yan, Shanshan Zhang, Zhaojian Gong, Guiyuan Li, Zhaoyang Zeng, Fuyan Wang, Can Guo, Wei Xiong

**Affiliations:** ^1^NHC Key Laboratory of Carcinogenesis and Hunan Key Laboratory of Cancer Metabolism, Hunan Cancer Hospital and The Affiliated Cancer Hospital of Xiangya School of Medicine, Central South University, Changsha, China; ^2^Key Laboratory of Carcinogenesis and Cancer Invasion of The Chinese Ministry of Education, Cancer Research Institute, Central South University, Changsha, China; ^3^Hunan Key Laboratory of Nonresolving Inflammation and Cancer, Disease Genome Research Center, The Third Xiangya Hospital, Central South University, Changsha, China; ^4^Department of Stomatology, Xiangya Hospital, Central South University, Changsha, China; ^5^Department of Oral and Maxillofacial Surgery, The Second Xiangya Hospital, Central South University, Changsha, China

**Keywords:** breast cancer, potassium channel, KCNK6, proliferation, invasion, migration

## Abstract

Breast cancer is the most common malignant tumor in women, and its incidence is increasing each year. To effectively treat breast cancer, it is important to identify genes involved in its occurrence and development and to exploit them as potential drug therapy targets. Here, we found that potassium channel subfamily K member 6 (KCNK6) is significantly overexpressed in breast cancer, however, its function in tumors has not been reported. We further verified that KCNK6 expression is upregulated in breast cancer biopsies. Moreover, overexpressed KCNK6 was found to enhance the proliferation, invasion, and migration ability of breast cancer cells. These effects may occur by weakening cell adhesion and reducing cell hardness, thus affecting the malignant phenotype of breast cancer cells. Our study confirmed, for the first time, that increased KCNK6 expression in breast cancer cells may promote their proliferation, invasion, and migration. Moreover, considering that ion channels serve as therapeutic targets for many small molecular drugs in clinical treatment, targeting KCNK6 may represent a novel strategy for breast cancer therapies. Hence, the results of this study provide a theoretical basis for KCNK6 to become a potential molecular target for breast cancer treatment in the future.

## Introduction

The latest global cancer data released by the international cancer research agency (IARC) of the World Health Organization, show that in 2020, the number of new breast cancer cases worldwide was as high as 2.26 million, with an approximated 680,000 associated female deaths, far exceeding that of any other female cancer. In fact, female breast cancer surpassed lung cancer to become the most commonly diagnosed cancer worldwide. It is estimated that at least 1 million new breast cancer patients will be diagnosed by 2040 (The, 2018). Currently, with the popularization of tumor screening and detection technology, as well as the improvements in comprehensive treatment strategies, a variety of treatment methods for breast cancer have been developed that benefit the majority of patients. However, 20% of patients will develop metastatic breast cancer, the prognosis of which is generally poor. Furthermore, 90% of patients may die as a result of recurrence and metastasis (Ali and Coombes, 2002; Rabbani and Mazar, 2007; Makki, 2015). With the development of modern oncology, the unrestricted proliferation of tumor cells, leading to their invasion and metastasis, has been confirmed as the fundamental cause of breast cancer progression and patient death (Chaffer et al., 2016; Mittal, 2018). It is, therefore, of urgency to predict the biological behavior of breast cancer by studying the proliferation, invasion, and metastasis of cancer cells, while also seeking to identify genes and potential targets involved in the occurrence and development of breast cancer.

A large number of studies have demonstrated that the progression of breast cancer is closely related to a variety of potassium channels, as the overexpression of many such channels has been reported in breast cancer (Hemmerlein et al., 2006; Dookeran et al., 2017), and found to be related to the poor prognosis of patients (Brevet et al., 2009b; Oeggerli et al., 2012; Faouzi et al., 2016). Potassium channel subfamily K member 6 (KCNK6) is a background potassium channel belonging to the double-pore domain potassium channel family, that facilitates the leakage of potassium ions out of cells primarily by regulating the resting membrane potential and life process of cells. Although KCNK6 is reportedly overexpressed in breast cancer cells (Williams et al., 2013), its role in malignant progression of tumors, has not been reported. Due to various unique characteristics of potassium channels, their altered expression may be useful for diagnosis and treatment. Indeed, Kv1.3 (Mohr et al., 2019), Kv10.1 (García-Quiroz et al., 2019), and Kv11.1 (Wang et al., 2015; Breuer et al., 2019) have proven to be potential molecular targets for breast cancer therapy. We, therefore, sought to verify the expression of KCNK6 in breast cancer and detect its function in the malignant phenotype to explore its potential as a new therapeutic target for breast cancer.

Recent studies have shown that mechanical factors (cell hardness, adhesion, etc.) play a key role in regulating the structure and function of cells, particularly in the process of cell carcinogenesis (Mierke, 2014; Mierke, 2019; Nia et al., 2020; Shen et al., 2020). As has been well-documented, during the process of metastasis, cancer cells must pass through a series of barriers, including the basement membrane, extracellular matrix, vascular wall, etc., requiring them to have strong invasive and migratory capacity (Friedl and Alexander, 2011; Trepat et al., 2012; Mittal, 2018). Many studies have shown that the occurrence of these cellular behaviors is often accompanied by a stronger deformability, facilitating the successful breaching of these barriers, that is, cells must become softer and more likely to detach from surrounding tissues (Cross et al., 2007; Rubiano et al., 2018). The adhesive properties of tumor cells must also, therefore, become weaker (Friedl and Wolf, 2003; Hamidi and Ivaska, 2018). Hence, the comprehensive consideration of a number of individual factors, including cell adhesion and hardness, can provide new insights to further our understanding regarding the mechanism of malignant tumor progression.

In the current study, we downloaded two groups of breast cancer genome-wide expression microchips from the Gene Expression Omnibus (GEO) database. Through data mining of the microchip dataset, we found that the expression of KCNK6 was significantly increased in breast cancer. Through analysis of TCGA data, it was found that the upregulation of KCNK6 is associated with malignant progression of breast cancer. We further verified, via immunohistochemistry, that KCNK6 expression is significantly upregulated in breast cancer and may lead to enhanced proliferation and invasion in breast cancer cell lines. Meanwhile, KCNK6 knockdown reversed these effects. Further, we found that high levels of KCNK6 decrease the adhesion and hardness of breast cancer cells, while its knockdown increased these properties, thereby affecting their biophysical phenotypes.

## Materials and Methods

### Clinical Samples

In this study, three sets of expression microarrays published online in the GEO public database were used to analyze the data: GSE42568 (including 17 cases of normal breast tissue and 104 cases of breast cancer tissue), GSE10780 (including 143 cases of normal breast tissue and 42 cases of breast cancer tissue) and GSE53752 (including 25 cases of normal breast tissue and 51 cases of breast cancer tissue).

Breast cancer tissue samples were provided by Xiangya Second Hospital of Central South University. The samples included 16 pairs of paraffin-embedded breast cancer tissue sections and non-tumor breast tissue sections, which were used to detect the expression of KCNK6 protein by immunohistochemistry. The clinical information of the patients from whom the sections were obtained is shown in Table 1. The use of all samples was authorized by the Ethics Committee of Central South University, and informed consent was obtained from all patients.

**TABLE 1:** Clinical patient information KCNK6 expression in breast cancer tissues and corresponding adjacent tissues of paraffin-embedded tissue sections.

**Patient No.**	**Age**	**T**	**N**	**M**	**Clinic**	**The expression of KCNK6 in the normal sample**	**The expression of KCNK6 in the turmor sample**
Pat 01	52	T2	N0	M0	IIa	0	9
Pat 02	50	T2	N0	M0	IIa	0	3
Pat 03	47	T2	N0	M0	IIa	2	6
Pat 04	53	T2	N1	M0	IIb	2	9
Pat 05	63	T2	N1	M0	IIb	1	6
Pat 06	50	T2	N1	M0	IIb	0	6
Pat 07	64	T2	N0	M0	IIa	4	9
Pat 08	49	T2	N0	M0	IIa	0	3
Pat 09	53	T2	N1	M0	IIb	0	9
Pat 10	53	T2	N0	M0	IIa	0	6
Pat 11	74	T1c	N1	M0	IIa	2	9
Pat 12	58	T2	N1	M0	IIb	0	6
Pat 13	49	T1c	N0	M0	II	1	3
Pat 14	50	T2	N0	M0	IIa	0	6
Pat 15	51	T2	N2a	M0	IIIa	1	9
Pat 16	44	T2	N1	M0	IIb	4	9

*The age, TNM stage and tumor grade of each patient are presented, and the immunohistochemical results of breast cancer and corresponding paracancerous tissues were interpreted to show the expression of KCNK6. The immunohistochemical scoring principle: according to the strength of KCNK6 signal, light yellow was scored 1, brown was 2, brown and non-specific staining was 3. According to the percentage of tumor cells with KCNK6 signaling relative to all tumor cells in the section: 0–25% was assigned a score of 1, 25% was 2, 50% was 3, and 75% was 4; the product of the two represented the final score. If the final score was 0, KCNK6 was judged as negative expression; if the final score was ≤3, KCNK6 was judged as low expression; if the final was >3, KCNK6 was judged as high expression.*

### KCNK6 Overexpression and Knockdown in Breast Cancer Cell Lines

The breast cancer cell lines, MDA-MB-231 and MCF-7, used in this study were preserved in the Molecular Genetics Room of the Institute of Oncology, Central South University. Cells were placed in complete Dulbecco’s modified eagle medium (DMEM) containing 10% fetal bovine serum (FBS) and 1% penicillin/streptomycin. Cells were cultured in a 37°C incubator containing 5% CO_2_.

The pcDNA6/myc-His C vector was employed as the control empty vector, as well as the KCNK6 overexpression vector. After annealing, the CDS fragment of KCNK6 was inserted into the vector by matching the viscous ends of EcoRI and NotI to obtain the overexpression vector.

The control empty vector of the shRNA vector is PLVshRNA-EGFP_(2A) Puro, provided by Inovogen company. After annealing, the shRNA fragment was inserted into the vector by matching the viscous ends of the EcoRI and BamHI sites to produce the shKCNK6 lentiviral vector.

The two shRNA sequences used to target KCNK6 are as follows: shRNA-1: 5′-GATCCGCAGGCAGGAAACAGACATAT TCAAGAGATATGTCTGTTTCCTGCCTGTTTTTT-3′ and shRNA-2: 5′-GATCCGCCCTTAACCATGACACCATTTCAAG AGAATGGTGTCATGGTTAAGGGTTTTTT-3′.

For overexpression KCNK6, breast cancer cells were plated overnight and transfected with the overexpression KCNK6 vector or control empty vector using the Neofect transfection reagent (Invitrogen, Carlsbad, CA, United States) in OptiDMEM medium (Invitrogen). Alternatively, 293T cells were transfected in the same way with either the shRNA or empty control vectors in the same way and incubated. The culture medium was harvested after 60h. The virus particles in the culture medium, containing the shRNA, were then used to infect breast cancer cell lines. The cells with KCNK6 successfully knocked down were screened with puromycin.

### RNA Extraction and Quantitative PCR Assay

The cellular RNA was extracted using Trizol reagent (Invitrogen) and reverse transcribed into cDNA by Quantscript RT kit (abm, Richmond, BC, Canada). KCNK6 specific primers were used for real-time quantitative polymerase chain reaction (PCR). A 5 × All-In-OneMasterMix kit (abm, Richmond, BC, Canada) was used for real-time quantitative PCR. A CFX96 real-time PCR detection system (Bio-Rad, Hercules, CA, United States) was used to detect the relative expression level of KCNK6. The sequence of qPCR primers was as follows: KCNK6-F, 5′-CTAAACCCCTCCTGTGTGCT-3′; KCNK6-R, 5′-CAACACCTCACCTCCTCCAT-3′; GAPDH-F, 5′-CAACGGATTTGGTCGTATTGG-3′; and GAPDH-R, 5′-TGACGGTGCCATGGA ATTT-3′.

### Western Blotting

Whole cell lysates were obtained using RIPA buffer (beyotime, China) and centrifugation at. The BCA protein analysis kit (Pierce, Grand Island, NY, United States) was used to determine protein concentration. A total of 50 μg of cell lysate was separated by 10% sodium dodecyl sulfate-polyacrylamide gel electrophoresis, transferred to a polyvinylidene fluoride (PVDF) membrane (Millipore, Billerica, MA, United States) and blocked with 5% skim milk for 2 h at room temperature. The membrane was then incubated with primary antibodies (Cusabio, Zhengzhou, Henan, CHINA) at 4°C overnight and washed with 1 × PBST the next day. Second antibodies coupled with horseradish peroxidase was then added to the membranes and incubated at room temperature for 2 h. The signal was detected with an ECL detection reagent.

### MTT Assays

MTT assays were used to detect cell proliferation ability. Firstly, the cells in the logarithmic growth phase were digested and re-suspended to prepare cell suspension, and 800 cells per well were inoculated into a 96-well plate. At least five parallel wells were used for each group for 6 days. When the cells adhered to the wall, 20 μl MTT solution was added to each well. The plate was then placed into the culture box for further culture for 4 h. Two-hundred microliters of DMSO was added to each well, and the plate was placed on the shaking table for 10 min. The absorbance of the sample at a wavelength of 490 nm was detected with a multi-function enzyme labeling instrument. The OD value was plotted with the time point as the abscissa and OD value as the ordinate.

### Clone Formation Assays

Clone formation assays were used to detect cell proliferation ability. The cells in the logarithmic growth phase were digested, centrifuge, add the culture medium and re- suspended, and blow into a single cell. The cells were then inoculated in a 12-well plate with 1,500 cells per well. When a naked eye-visible monoclonal (no less than 50 cells per clone) was formed, the culture was removed from the plate and the plate was rinsed with PBS and fixed with 4% paraformaldehyde for 15 min. This was followed by staining with crystal violet for 15 min. Crystal violet was then carefully washed. The 12-well plate was scanned, and the number of clones in each group was counted with ImagePro Plus 6.0 software.

### Transwell Cell Migration and Invasion Assays

Transwell Cell Culture Inserts (8-μm pore size, BD Biosciences, Franklin Lakes, NJ, United States) was used to evaluate the migration and invasive ability of the cells. The melted BD Matrigel, was mixed with the serum-free medium in the ratio of V Matrigel :V culture medium = 1:9. 20 μl was added to each transwell chamber and put into an incubator at 37°C for 2 h, and the matrix glue was condensed in the chamber. Note: this step is not required for the Transwell migration experiment. Two-hundred microliters of serum-free medium containing 3 × 104 cells were added to the upper chamber of 24-well plates. Eight-hundred microliters of culture medium containing 20% FBS was then added, and the plate was placed into the Transwell chamber and soaked. The plate was then incubated at 37°C until the cells are observed to be falling to the bottom of the plate. The plate was then removed from the chamber, washed twice with normal saline, fixed with 4% paraformaldehyde, and stained with 0.5% crystal violet. Wiped off the matrigel glue and the cells on the upper surface of the chamber. Five visual fields were randomly selected to for imaging under the inverted microscope. The invaded cells were counted using ImageProPlus 6.0 software.

### Scratch Healing Assays

Scratch healing assays were used to observe cell migration ability. The cells were inoculated in a 6-well plate and cultured in an incubator at 37°C until the cells cover the whole hole. The tip of the p200 straw was crossed perpendicular to the opening of the well (three lines could be divided into four equal lines in horizontal and vertical directions). Then, DMEM with 2% FBS was added for cell culture, and 5% hydroxyurea was added to inhibit cell division. The culture group and hydroxyurea were replaced at 0 and 24 h, respectively. The scratches were observed and photographed under the microscope until fully healed. The width of scratches at different time points was measured by ImagePro Plus 6.0 software.

### Atomic Force Microscopy (AFM)

A single MDA-MB-231 cell was measured with an atomic force microscope (JPK NanoWizard 4 BioScience, JPK Instruments, Berlin, Germany). The probe used was a 100 μm V-shaped probe μm (HYDRA6V-100NG, AppNano, Mountain View CA, United States). The indentation material was a silicon nitride cantilever with a spring constant of 0.292 N/m. Indentation was carried out at a loading and retraction speed of approximately 2.5 μm/s for all experiments to avoid the viscosity effect. The cells in the Petri dish were measured at room temperature. First, the cells were adhered to the Petri dish, followed by washing three times with PBS. The cells were fixed with 2% glutaraldehyde for 45 s, followed by fixation with 4% polymethanol for 20 min. Finally, the cells were washed with PBS more than 5 times. The cells were then covered with an appropriate amount of PBS, and with an AFM. In order to better simulate the physiological deformation of cells, the indentation depth was selected to be at least 1 mm. QI mode was used for imaging, and JPK software was used to analyze the images obtained by scanning.

### Statistical Analysis

Statistical analyses were carried out with GraphPad Prism 9.0 software (GraphPad, San Diego, CA, United States). For immunohistochemical statistical analysis, a paired Student’s *t*-test was used to evaluate the significant difference between the two groups of data. All other analyses were conducted using unpaired *t*-tests to evaluate the significant difference. *P* < 0.05 was considered to indicate a statistically significant difference. All data are shown as the mean ± standard deviation of at least three independent experiments.

## Results

### KCNK6 Expression Is Significantly Increased in Breast Cancer

**FIGURE 1:**
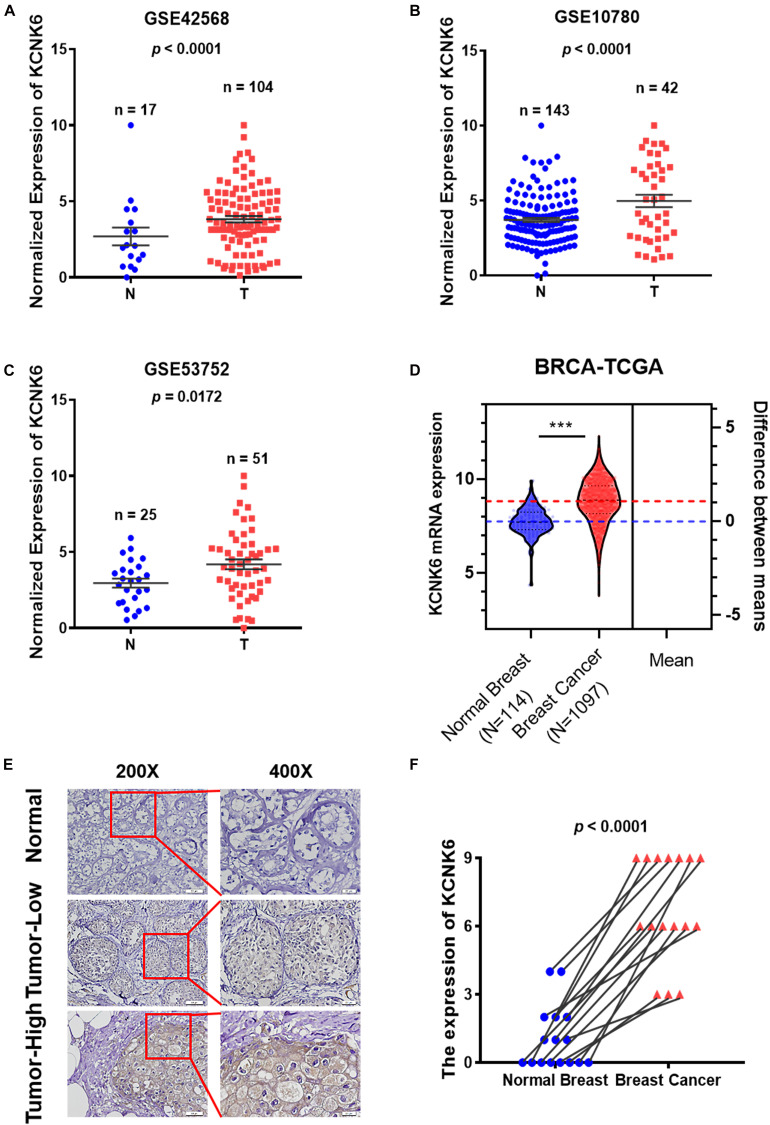
Potassium channel protein KCNK6 expression was significantly increased in breast cancer. **(A)** KCNK6 expression was analyzed using the gene expression microarray data set GSE42568 from the Gene Expression Omnibus (GEO) database. Compared with normal breast tissue, KCNK6 expression in breast cancer tissue was significantly higher. N denotes normal, T, denotes tumor. **(B)** KCNK6 expression was analyzed using the gene expression microarray data set GSE10780 from the GEO database. Compared with normal breast tissue, KCNK6 expression in breast cancer tissue was significantly higher. **(C)** KCNK6 expression was analyzed using the gene expression microarray data set GSE53752 from the GEO database. Compared with normal breast tissue, KCNK6 expression in breast cancer tissue was significantly higher. **(D)** Using TCGA database to analyze the expression of KCNK6. **(E)** KCNK6 expression in normal breast tissue and breast cancer tissue. **(F)** KCNK6 expression in 16 pairs of normal breast tissues and breast cancer tissues. ***P < 0.001.

To identify differentially expressed ion channel proteins in breast cancer, we downloaded the gene expression profile data for two groups of breast cancer tissues from the GEO database: GSE42568, GSE10780, and GSE53752. The expression profiles of each member of the common ion channel family were analyzed. We found that the expression of potassium channel protein KCNK6 in normal breast tissue was significantly lower than that in breast cancer tissue (*P* < 0.05; Figures 1A–C). The breast cancer data downloaded from the TCGA database were further processed with python. Compared with normal breast tissues, the expression of KCNK6 was significantly upregulated in breast cancer tissue (^∗∗∗^*P* < 0.001; Figure 1D).

Furthermore, we collected paraffin sections of cancerous tissues and corresponding paracancerous tissues from 16 patients with breast cancer (Table 1). We then detected the expression of KCNK6 expression in clinical breast cancer samples via immunohistochemistry. The results showed that the expression level of KCNK6 was significantly higher in breast cancer tissues than in normal breast tissues (*P* < 0.0001; Figures 1E,F).

### KCNK6 Expression Affects the Proliferation of Breast Cancer Cells

**FIGURE 2:**
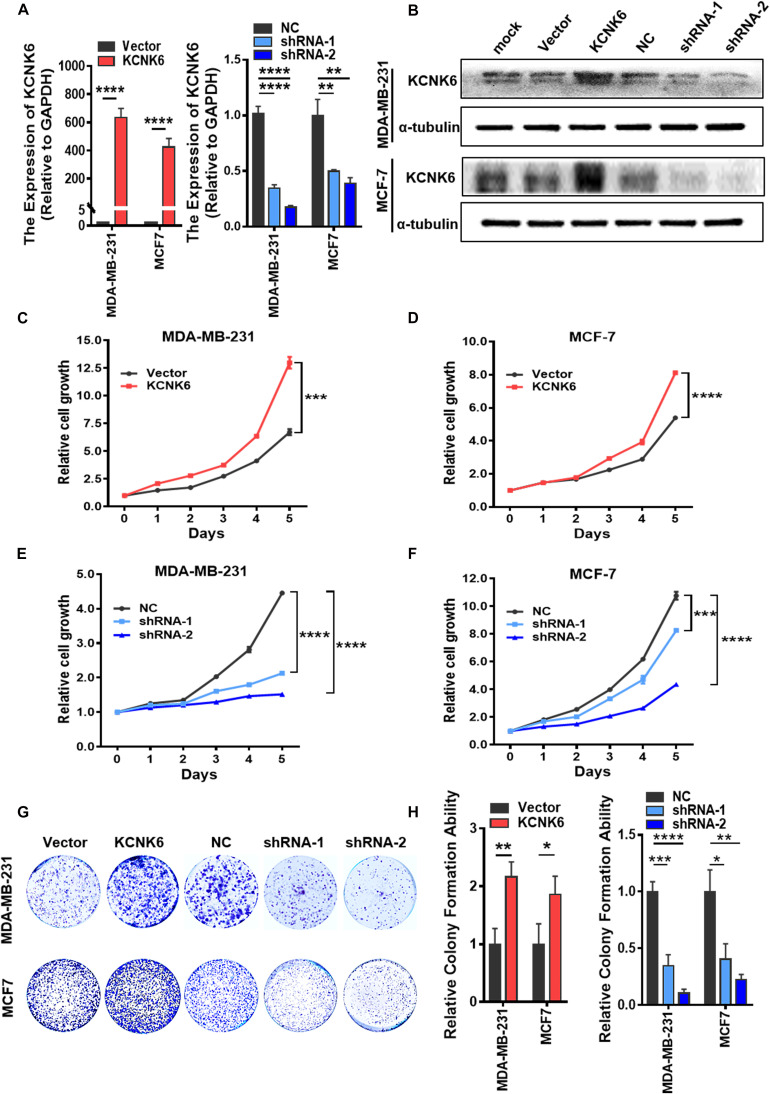
KCNK6 expression affects breast cancer cell proliferation. **(A)** qRT-PCR assay was used to detect the overexpression and knockdown efficiency of KCNK6 in MDA-MB-231 cells and MCF-7 cells. **(B)** Western blot assay was used to detect the overexpression and knockdown efficiency of KCNK6 in MDA-MB-231 cells and MCF-7 cells. **(C)** MTT experiment showed that overexpression of KCNK6 could significantly promote the proliferation of MDA-MB-231 cells. **(D)** MTT experiment showed that overexpression of KCNK6 could significantly promote the proliferation of MCF-7 cells. **(E)** MTT experiment showed that knocking down KCNK6 could significantly inhibit the proliferation of MDA-MB-231 cells. **(F)** MTT experiment showed that knocking down KCNK6 could significantly inhibit the proliferation of MCF-7 cells. **(G)** Colony formation assay showed that overexpression of KCNK6 could significantly promote the proliferation of MDA-MB-231 cells and MCF-7 cells, while knocking down KCNK6 could significantly inhibit the proliferation of MDA-MB-231 cells and MCF-7 cells. **(H)** Statistical analysis of the number of clones in each group showed that overexpression of KCNK6 could significantly promote the proliferation of MDA-MB-231 cells and MCF-7 cells, while knock down KCNK6 to get the opposite result. *P < 0.05; **P < 0.01; ***P < 0.001; ****P < 0.0001.

The high expression of the potassium channel protein KCNK6 in breast cancer suggests that it may be involved in the malignant transformation of breast cancer. We, therefore, designed further experiments to verify its biological function in breast cancer. First, we overexpressed KCNK6, in MDA-MB-231 and MCF-7 cell lines and used lentivirus vectors to infect cells with shRNA targeting KCNK6. A control group was also created by infecting cells with an empty vector. In this way, we successfully generated MDA-MB-231 cells and MCF-7 cells with stable KCNK6 knockdown. The results showed that considerable KCNK6 overexpression and knockdown were obtained (^∗∗^*P* < 0.01; ^****^P < 0.0001; Figures 2A,B).

Although the current definition of tumor characteristics is extensive, the most common and prominent feature of tumors is their unlimited proliferative capacity. Therefore, we designed experiments to explore whether the expression of KCNK6 affects breast cancer cell proliferation. Through an MTT assay, we showed that overexpression of KCNK6 significantly promoted the proliferation of MDA-MB-231 and MCF-7 cells (^∗∗∗^*P* < 0.001; ^****^P < 0.0001; Figures 2C,D), while its knockdown significantly inhibited MDA-MB-231 and MCF-7 cell proliferation compared with control cells (^∗∗∗^*P* < 0.001; ^****^P < 0.0001; Figures 2E,F). The results of a colony formation assay also showed that overexpression of KCNK6 promoted growth of MDA-MB-231 and MCF-7 cells. Meanwhile, the proliferative ability of MDA-MB-231 and MCF-7 cells stably transfected with shRNA was significantly weaker than that of MDA-MB-231 and MCF-7 cells transfected with the control empty vector (^∗^*P* < 0.05; ^∗∗^*P* < 0.01; ^∗∗∗^*P* < 0.001; ^****^P < 0.0001; Figures 2G,H).

### KCNK6 Expression Affects the Invasion and Migration of Breast Cancer Cells

**FIGURE 3:**
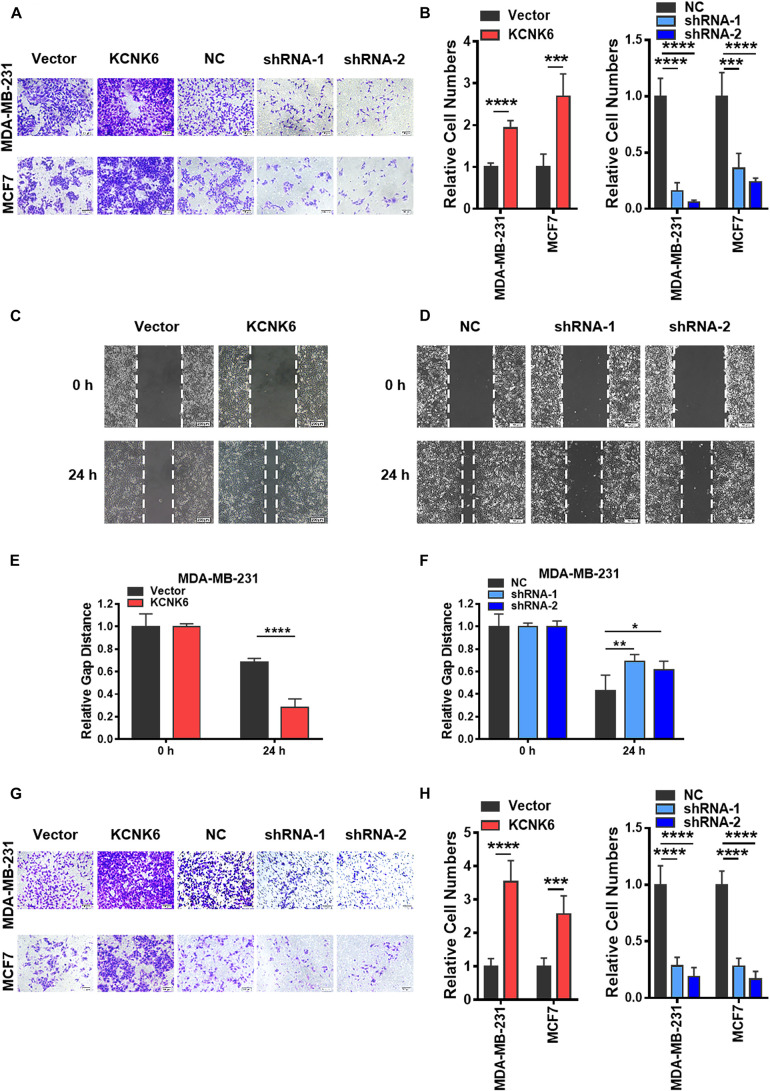
KCNK6 expression affects the invasion and migration of breast cancer cells. **(A)** Transwell cell migration experiment results showed that the migration ability of MDA-MB-231 cells and MCF-7 cells was significantly enhanced after overexpression of KCNK6, and the migration ability of MDA-MB-231 cells and MCF-7 cells was significantly inhibited after knocking down KCNK6. **(B)** The number of migrating cells in each group was counted and plotted, showing that overexpression of KCNK6 could significantly promote the migration of MDA-MB-231 cells and MCF-7 cells, while knocking down KCNK6 significantly inhibited the migration of MDA-MB-231 cells and MCF-7 cells. **(C)** The scratch healing experiment showed that overexpression of KCNK6 could significantly promote the migration of MDA-MB-231 cells. **(D)** The software was used to measure the changes of scratch healing in each group at different time points, and the statistical chart was drawn. The results showed that overexpression of KCNK6 could significantly promote the migration of MDA-MB-231 cells. **(E)** In the scratch healing experiment showed that knocking down KCNK6 could significantly inhibit the migration of MDA-MB-231 cells. **(F)** The software was used to measure the changes of scratch healing in each group at different time points, and the statistical chart was drawn. The results showed that knocking down KCNK6 could significantly inhibit the migration of MDA-MB-231 cells. **(G)** Transwell cell invasion experiment showed that the invasive ability of MDA-MB-231 cells and MCF-7 cells was significantly enhanced after overexpression of KCNK6, and the invasive ability of MDA-MB-231 cells and MCF-7 cells was significantly inhibited after knocking down KCNK6. **(H)** The number of invasive cells in each group was counted and plotted showing that the invasive ability of MDA-MB-231 cells and MCF-7 cells was significantly enhanced after overexpressing KCNK6. After knocking down KCNK6, the invasive ability of MDA-MB-231 cells and MCF-7 cells was significantly inhibited. *P < 0.05; **P < 0.01; ***P < 0.001; ****P < 0.0001.

A high invasive ability is also considered to be a significant feature of tumor cells. We further designed experiments to verify whether the expression level of KCNK6 affects breast cancer cell invasion and migration. Transwell cell migration assays demonstrated that the migratory capacity of MDA-MB-231 and MCF-7 cells overexpressing KNCK6 was significantly enhanced compared to the control group, while knocking the expression of KCNK6 down significantly inhibited this effect (^∗∗∗^*P* < 0.001; ^****^P < 0.0001; Figures 3A,B). The same result was obtained using a scratch healing experiment in the MDA-MB-231 cell line (^∗^*P* < 0.05; ^∗∗^*P* < 0.01; ^****^P < 0.0001; Figures 3C–F). Meanwhile, considering that MCF-7 cells are unable to grow in clusters under the 2D *in vitro* culture conditions, the scratch healing study could not be carried out. Simultaneously, we observed via transwell cell invasion assay results, that overexpression of KCNK6 also promotes the invasive ability of breast cancer cell lines compared to control cells; whereas the opposite effect was observed following KCNK6 knockdown (^∗∗∗^*P* < 0.001; ^****^P < 0.0001; Figures 3G,H).

### KCNK6 Expression Affects the Biophysical Properties of Breast Cancer Cells

**FIGURE 4:**
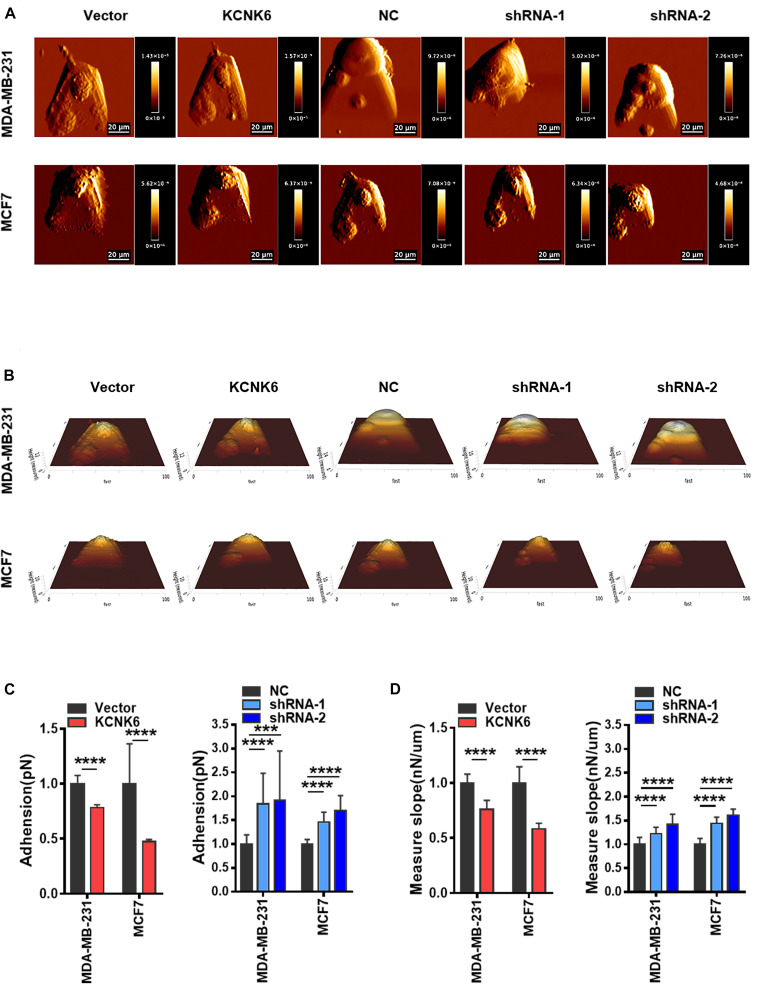
KCNK6 expression affects the biophysical properties of breast cancer cells. **(A)** Representative AFM deflection images of MDA-MB-231 cells and MCF-7 cells in control group, overexpression KNCK6 group and knocked down KCNK6 group. The cells of each group were fixed in a petri dish, and were selected randomly for detection by atomic force microscopy (AFM). **(B)** Representative three-dimensional height distribution map of MDA-MB-231 cells and MCF-7 cells in control group, overexpression KNCK6 group and knock down KCNK6 group, the detection method is the same as above. **(C)** Thirty cells were randomly selected form each group, and the adhesion was statistically analyzed and plotted based on JPK image processing software. High levels of KCNK6 could weaken the adhesion between MDA-MB-231 cells and MCF-7 cells, while low levels of KCNK6 increased the adhesion between cells. **(D)** Thirty cells were randomly selected from each group, and their stiffness was statistically analyzed and plotted based on JPK image processing software. Overexpression of KCNK6 significantly reduced the stiffness of MDA-MB-231 and MCF-7 cells, while knocking down KCNK6 could significantly increase the stiffness of cells. ***P < 0.001; ****P < 0.0001.

Recent studies have shown that the biophysical characteristics of cells are involved in regulating tumor cell occurrence and development (Mierke, 2019). AFM can be used to detect the mechanical properties of a single cell with strong spatial resolution and high force sensitivity. To clarify the effect of KCNK6 expression on the biophysical characteristics of cells, we used atomic force microscopy to analyze the cell morphology before and after altering KCNK6 expression. A representative AFM deflection image (Figure 4A) as well as a three-dimensional height distribution map (Figure 4B) of individual experimental cells and control cells are presented to demonstrate the surface characteristics of cells. We were then able to use the force curve obtained from each point in the detection map for further statistical analysis. The results showed that KCNK6 overexpression caused weakening of the cell adhesive properties, indicating improved detachment from the surrounding tissues and cells, reflecting the previously observed enhanced invasion and metastasis properties. Meanwhile, KCNK6 knockdown caused enhanced cell adhesion, indicating that cell invasion and metastasis would have decreased (^****^P < 0.0001; Figure 4C).

Current studies have also reported that the greater stiffness of cancer cells, the weaker the deformability and worse invasive ability. Atomic force microscopy showed that the stiffness of overexpressing KCNK6 cells decreased, indicating that the cells became softer, with increased deformability and invasiveness increased; whereas knocking down KCNK6, caused breast cancer cells to increase in stiffness, reflecting their weakened ability to invade or metastasize (^****^P < 0.0001; Figure 4D). Taken together, these results suggest that KCNK6 may affect a series of physiological activities in breast cancer cells, including cell invasion and metastasis, by altering their biophysical characteristics.

## Discussion

In this study, through the data mining of two groups of breast cancer gene expression profile microarray datasets, we found that the potassium channel protein KCNK6 was expressed at an abnormally high level in breast cancer cells. Additionally, in the collected clinical tissue samples, we found that KCNK6 expression was significantly higher in breast cancer tissue than in normal breast tissue. We further detected the biological function of KCNK6 in breast cancer cell lines and found that overexpression of KCNK6 could significantly enhance the proliferative, invasion, and migratory properties of MDA-MB-231 and MCF-7 cells. Moreover, we observed that the proliferative, invasive, and migratory capacity of breast cancer cells with KCNK6 knocked down was significantly weakened. These results suggest that KCNK6, as a potential biomarker, may play a key role in the occurrence, development, and prognosis of breast cancer.

According to the different modes of activation, ion channels are generally divided into voltage-gated, ligand-gated, and mechanical-gated channels. The term “ion channel” often refers to the voltage-gated channel. The opening of this channel is primarily controlled by the membrane potential, while channels are generally named after the ions that can most readily pass through them. These include the potassium, sodium, calcium, and chloride ion channels (Prevarskaya et al., 2018). Potassium channels are the most widely distributed and diverse class of voltage-gated ion channels, and are generally classified into four categories: voltage-gated potassium channels, calcium-activated potassium channels, inward-rectifier potassium channels, and two-pore-domain potassium channels. The potassium channel protein KCNK6 is associated with two-pore-domain potassium channels (Sigworth, 2001). KCNK6 is a member of the potassium channel superfamily and forms a complete potassium channel in the form of a dimer. Each subunit contains two pore domains, each of which two transmembrane regions consistent with the typical structural characteristics of two-pore-domain potassium channels (Kuang et al., 2015).

It is well-documented that various ion channels widely exist in various tissues and cells and play an important regulatory role in basic life processes (Huang and Jan, 2014; Pardo and Stühmer, 2014; Bates, 2015). The normal structure and function of ion channels are the basis for maintaining the life process of cells. Their most important biological function is to regulate the permeability of many kinds of ions and maintain osmotic pressure inside and outside the cell, serving as an important means for cells to exchange substances with their surroundings (Ding et al., 2007; Urrego et al., 2016; Assiri et al., 2019). Ion channels can directly affect a variety of biophysical properties, such as cell hardness, by regulating the osmotic pressure of cells. Therefore, based on their powerful functions, ion channels are usually used as therapeutic targets for a variety of small molecular drugs in clinical settings (Cannon, 2007; Bagal et al., 2013).

Potassium channels are encoded by approximately 77 genes (Pardo and Stühmer, 2014), and are the most widely studied class of ion channels. Their normal biological function is to specifically regulate the permeability of potassium ions and hinder the permeability of other ions according to environmental signals. These channels also maintain the normal membrane potential of cells and regulate osmotic pressure (Huang and Jan, 2014). Potassium channel proteins are widely involved in various physiological and pathological processes in cells (Schwab et al., 2012). Inhibiting KCNK6 expression in macrophages can inhibit the inflammatory response induced by inflammatory bodies (Di et al., 2018). KCNK6 is also highly expressed in the vascular system, when its abnormally low expression may lead to vascular dysfunction (Lloyd et al., 2011) and pulmonary hypertension (Pandit et al., 2014). Studies on the nervous system have found that abnormal KCNK6 expression may be associated with pain caused by inflammation (Marsh et al., 2012). More importantly, potassium channels show high variability and abnormal expression in many tumor types (Cho et al., 2006; Brevet et al., 2009a; Menéndez et al., 2012), such as breast cancer (Ko et al., 2013), colorectal cancer (Ishaque et al., 2018), prostate cancer (Rose et al., 2018), lung cancer (Zhang et al., 2017), liver cancer (Wang et al., 2017), and glioma (Huang et al., 2015).

Many studies have shown that potassium channels, including KCNN4 (Steudel et al., 2017), KCNA1 (Lallet-Daher et al., 2013), Kv11.1 (Breuer et al., 2019), KCNK9 (Mu et al., 2003; Sun et al., 2016), KCNE1 (Becchetti et al., 2017), and GIRK1 (Stringer et al., 2001) are involved in the regulation of malignant breast cancer transformation. Although it has been reported that KCNK6 expression is increased in breast cancer (Williams et al., 2013) and thyroid carcinoma (Lin et al., 2020), its function had not been previously reported. Therefore, our study revealed, for the first time, that altered KCNK6 expression in breast cancer cell lines results in significant changes in cell adhesion and hardness, leading us to postulate that its expression will also affect the flow of potassium ions as well as the liquid flow, thereby directly impacting the cell hardness and adhesion. Thus, KCNK6 may participate in regulating proliferation, invasion, and migration in breast cancer cells. It is, therefore, suggested that the development of specific small molecular therapeutic drugs for targeting KCNK6 may offer potential for treating diseases, including breast cancer. KCNK6 expression is increased in breast cancer, and its correlation with clinical progression and breast cancer prognosis is worthy of further study. We have reason to believe that through more in-depth and detailed investigations, KCNK6 may become a new biomarker of breast cancer.

In short, we found that the potassium channel protein KCNK6 is significantly overexpressed in breast cancer and that its expression level significantly affects the proliferation, invasion and migration capacity of breast cancer cells, accompanied by changes in their biophysical characteristics. This suggests that more detailed analysis of KCNK6 will help to clarify the pathogenesis of breast cancer, identify new therapeutic targets for breast cancer, and promote development of new tumor therapy strategies.

## Data Availability Statement

The original contributions presented in the study are included in the article/Supplementary Material, further inquiries can be directed to the corresponding author/s.

## Author Contributions

XH, LT, XL, FX, YM, XJ, XD, MP, PW, MZ, JO, and LS collected the related manuscript and finished the manuscript and figures. WX, ZZ, and CG gave constructive guidance and made critical revisions. YH, QY, SZ, ZG, GL, and FW participated in the design of this review. All authors read and approved the final manuscript.

## Conflict of Interest

The authors declare that the research was conducted in the absence of any commercial or financial relationships that could be construed as a potential conflict of interest.

## References

[B1] Ali S. Coombes R. C. (2002). Endocrine-responsive breast cancer and strategies for combating resistance. *Nat. Rev. Cancer* 2 101–112. 10.1038/nrc721 12635173

[B2] Assiri A. A. Mourad N. Shao M. Kiel P. Liu W. Skaar T. C. (2019). MicroRNA 362-3p reduces hERG-related current and inhibits breast cancer cells proliferation. *Cancer Genom. Proteomics* 16 433–442. 10.21873/cgp.20147 31659098 PMC6885367

[B3] Bagal S. K. Brown A. D. Cox P. J. Omoto K. Owen R. M. Pryde D. C. (2013). Ion channels as therapeutic targets: a drug discovery perspective. *J. Med. Chem.* 56 593–624. 10.1021/jm3011433 23121096

[B4] Bates E. (2015). Ion channels in development and cancer. *Annu. Rev. Cell. Dev. Biol.* 31 231–247. 10.1146/annurev-cellbio-100814-125338 26566112

[B5] Becchetti A. Crescioli S. Zanieri F. Petroni G. Mercatelli R. Coppola S. (2017). The conformational state of hERGchannels determines integrin association, downstream signaling, and cancer progression. *Sci. Signal* 10:eaaf3236. 10.1126/scisignal.aaf3236 28377405

[B6] Breuer E. K. Fukushiro-Lopes D. Dalheim A. Burnette M. Zartman J. Kaja S. (2019). Potassium channel activity controls breast cancer metastasis by affecting β-catenin signaling. *Cell Death Dis.* 10:180. 10.1038/s41419-019-1429-0 30792401 PMC6385342

[B7] Brevet M. Fucks D. Chatelain D. Regimbeau J. M. Delcenserie R. Sevestre H. (2009a). Deregulation of potassium channels in pancreas adenocarcinomas: implication of KV1.gene promoter methylation. *Pancreas* 38 649–654. 10.1097/MPA.0b013e3181a56ebf 19465885

[B8] Brevet M. Haren N. Sevestre H. Merviel P. Ouadid-Ahidouch H. (2009b). DNA methylation of K(v)1.potassium channel gene promoter is associated with poorly differentiated breast adenocarcinoma. *Cell. Physiol. Biochem. Int. J. Exp. Cell. Physiol. Biochem. Pharmacol.* 24 25–32. 10.1159/000227810 19590190

[B9] Cannon S. C. (2007). Physiologic principles underlying ion channelopathies. *Neurotherapeutics* 4 174–183. 10.1016/j.nurt.2007.01.015 17395127

[B10] Chaffer C. L. San Juan B. P. Lim E. Weinberg R. A. (2016). EMT, cell plasticity and metastasis. *Cancer Metastasis Rev.* 35 645–654. 10.1007/s10555-016-9648-7 27878502

[B11] Cho Y. G. Kim C. J. Song J. H. Rhie D. J. Park Y. K. Kim S. Y. (2006). Genetic and expression analysis of the KCNRG gene in hepatocellular carcinomas. *Exp. Mol. Med.* 38 247–255. 10.1038/emm.2006.30 16819283

[B12] Cross S. E. Jin Y. S. Rao J. Gimzewski J. K. (2007). Nanomechanical analysis of cells from cancer patients. *Nat. Nanotechnol.* 2 780–783. 10.1038/nnano.2007.388 18654431

[B13] Di A. Xiong S. Ye Z. Malireddi R. K. S. Kometani S. Zhong M. (2018). The TWIK potassium efflux channel in macrophages mediates NLRP inflammasome-induced inflammation. *Immunity* 49 56.e–65.e. 10.1016/j.immuni.2018.04.032 29958799 PMC6051907

[B14] Ding X. W. Luo H. S. Jin X. Yan J. J. Ai Y. W. (2007). Aberrant expression of eagpotassium channels in gastric cancer patients and cell lines. *Med. Oncol.* 24 345–350. 10.1007/s12032-007-0015-y 17873312

[B15] Dookeran K. A. Zhang W. Stayner L. Argos M. (2017). Associations of two-pore domain potassium channels and triple negative breast cancer subtype in the cancer genome atlas: systematic evaluation of gene expression and methylation. *BMC Res. Notes* 10:475. 10.1186/s13104-017-2777-4 28899398 PMC5596847

[B16] Faouzi M. Hague F. Geerts D. Ay A. S. Potier-Cartereau M. Ahidouch A. (2016). Functional cooperation between KCa3.and TRPC channels in human breast cancer: role in cell proliferation and patient prognosis. *Oncotarget* 7 36419–36435. 10.18632/oncotarget.9261 27183905 PMC5095010

[B17] Friedl P. Alexander S. (2011). Cancer invasion and the microenvironment: plasticity and reciprocity. *Cell* 147 992–1009. 10.1016/j.cell.2011.11.016 22118458

[B18] Friedl P. Wolf K. (2003). Tumour-cell invasion and migration: diversity and escape mechanisms. *Nat. Rev. Cancer* 3 362–374. 10.1038/nrc1075 12724734

[B19] García-Quiroz J. García-Becerra R. Santos-Cuevas C. Ramírez-Nava G. J. Morales-Guadarrama G. árdenas-Ochoa N. C. (2019). Synergistic antitumorigenic activity of calcitriol with curcumin or resveratrol is mediated by angiogenesis inhibition in triple negative breast cancer xenografts. *Cancers* 11:1739. 10.3390/cancers11111739 31698751 PMC6896056

[B20] Hamidi H. Ivaska J. (2018). Every step of the way: integrins in cancer progression and metastasis. *Nat. Rev. Cancer* 18 533–548. 10.1038/s41568-018-0038-z 30002479 PMC6629548

[B21] Hemmerlein B. Weseloh R. M. Mello de Queiroz F. Knötgen H. Sánchez A. Rubio M. E. (2006). Overexpression of eagpotassium channels in clinical tumours. *Mol. Cancer* 5:41. 10.1186/1476-4598-5-41 17022810 PMC1621079

[B22] Huang X. Jan L. Y. (2014). Targeting potassium channels in cancer. *J. Cell Biol.* 206 151–162. 10.1083/jcb.201404136 25049269 PMC4107787

[B23] Huang X. He Y. Dubuc A. M. Hashizume R. Zhang W. Reimand J. (2015). EAG potassium channel with evolutionarily conserved function as a brain tumor target. *Nat. Neurosci.* 18 1236–1246. 10.1038/nn.4088 26258683 PMC4639927

[B24] Ishaque N. Abba M. L. Hauser C. Patil N. Paramasivam N. Huebschmann D. (2018). Whole genome sequencing puts forward hypotheses on metastasis evolution and therapy in colorectal cancer. *Nat. Commun.* 9:4782. 10.1038/s41467-018-07041-z 30429477 PMC6235880

[B25] Ko J. H. Ko E. A. Gu W. Lim I. Bang H. Zhou T. (2013). Expression profiling of ion channel genes predicts clinical outcome in breast cancer. *Mol. Cancer* 12:106. 10.1186/1476-4598-12-106 24053408 PMC3849355

[B26] Kuang Q. Purhonen P. Hebert H. (2015). Structure of potassium channels. *Cell. Mol. Life Sci.* 72 3677–3693. 10.1007/s00018-015-1948-5 26070303 PMC4565861

[B27] Lallet-Daher H. Wiel C. Gitenay D. Navaratnam N. Augert A. Le Calvé B. (2013). Potassium channel KCN amodulates oncogene-induced senescence and transformation. *Cancer Res.* 73 5253–5265. 10.1158/0008-5472.Can-12-3690 23774215

[B28] Lin X. Wu J. F. Wang D. M. Zhang J. Zhang W. J. Xue G. (2020). The correlation and role analysis of KCNK2/4/5/in human papillary thyroid carcinoma microenvironment. *J. Cancer* 11 5162–5176. 10.7150/jca.45604 32742463 PMC7378911

[B29] Lloyd E. E. Crossland R. F. Phillips S. C. Marrelli S. P. Reddy A. K. Taffet G. E. (2011). Disruption of K(2P)6.produces vascular dysfunction and hypertension in mice. *Hypertension* 58 672–678. 10.1161/hypertensionaha.111.175349 21876070 PMC3205080

[B30] Makki J. (2015). Diversity of breast carcinoma: histological subtypes and clinical relevance. *Clin. Med. Insights Pathol.* 8 23–31. 10.4137/CPath.S31563 26740749 PMC4689326

[B31] Marsh B. Acosta C. Djouhri L. Lawson S. N. (2012). Leak K^+^ channel mRNAs in dorsal root ganglia: relation to inflammation and spontaneous pain behaviour. *Mol. Cell Neurosci.* 49 375–386. 10.1016/j.mcn.2012.01.002 22273507 PMC3334831

[B32] Menéndez S. T. Villaronga M. A. Rodrigo J. P. Alvarez-Teijeiro S. García-Carracedo D. Urdinguio R. G. (2012). Frequent aberrant expression of the human ether à go-go (hEAG1) potassium channel in head and neck cancer: pathobiological mechanisms and clinical implications. *J. Mol. Med. (Berl)* 90 1173–1184. 10.1007/s00109-012-0893-0 22466864

[B33] Mierke C. T. (2014). The fundamental role of mechanical properties in the progression of cancer disease and inflammation. *Rep. Progr. Physics. Physical. Soc. (Great Britain)* 77:076602. 10.1088/0034-4885/77/7/076602 25006689

[B34] Mierke C. T. (2019). The matrix environmental and cell mechanical properties regulate cell migration and contribute to the invasive phenotype of cancer cells. *Rep. Progr. Physics. Physical. Soc. (Great Britain)* 82:064602. 10.1088/1361-6633/ab1628 30947151

[B35] Mittal V. (2018). Epithelial mesenchymal transition in tumor metastasis. *Annu. Rev. Pathol.* 13 395–412. 10.1146/annurev-pathol-020117-043854 29414248

[B36] Mohr C. J. Gross D. Sezgin E. C. Steudel F. A. Ruth P. Huber S. M. (2019). K(Ca)3.channels confer radioresistance to breast cancer cells. *Cancers* 11:1285. 10.3390/cancers11091285 31480522 PMC6770875

[B37] Mu D. Chen L. Zhang X. See L. H. Koch C. M. Yen C. (2003). Genomic amplification and oncogenic properties of the KCNK potassium channel gene. *Cancer Cell* 3 297–302. 10.1016/s1535-6108(03)00054-0 12676587

[B38] Nia H. T. Munn L. L. Jain R. K. (2020). Physical traits of cancer. *Science* 370:eaaz0868. 10.1126/science.aaz0868 33122355 PMC8274378

[B39] Oeggerli M. Tian Y. Ruiz C. Wijker B. Sauter G. Obermann E. (2012). Role of KCNMAin breast cancer. *PLoS One* 7:e41664. 10.1371/journal.pone.0041664 22899999 PMC3416802

[B40] Pandit L. M. Lloyd E. E. Reynolds J. O. Lawrence W. S. Reynolds C. Wehrens X. H. (2014). TWIK-channel deficiency leads to pulmonary hypertension through a rho-kinase-mediated process. *Hypertension* 64 1260–1265. 10.1161/hypertensionaha.114.03406 25245387 PMC4231005

[B41] Pardo L. A. Stühmer W. (2014). The roles of K(+) channels in cancer. *Nat. Rev. Cancer* 14 39–48. 10.1038/nrc3635 24336491

[B42] Prevarskaya N. Skryma R. Shuba Y. (2018). Ion channels in cancer: are cancer hallmarks oncochannelopathies? *Physiol. Rev.* 98 559–621. 10.1152/physrev.00044.2016 29412049

[B43] Rabbani S. A. Mazar A. P. (2007). Evaluating distant metastases in breast cancer: from biology to outcomes. *Cancer Metastasis Rev.* 26 663–674. 10.1007/s10555-007-9085-8 17823779

[B44] Rose A. M. Krishan A. Chakarova C. F. Moya L. Chambers S. K. Hollands M. (2018). MSRrepeats modulate gene expression and affect risk of breast and prostate cancer. *Ann. Oncol.* 29 1292–1303. 10.1093/annonc/mdy082 29509840

[B45] Rubiano A. Delitto D. Han S. Gerber M. Galitz C. Trevino J. (2018). Viscoelastic properties of human pancreatic tumors and in vitro constructs to mimic mechanical properties. *Acta biomaterialia* 67 331–340. 10.1016/j.actbio.2017.11.037 29191507 PMC5797706

[B46] Schwab A. Fabian A. Hanley P. J. Stock C. (2012). Role of ion channels and transporters in cell migration. *Physiol. Rev.* 92 1865–1913. 10.1152/physrev.00018.2011 23073633

[B47] Shen Y. Wang X. Lu J. Salfenmoser M. Wirsik N. M. Schleussner N. (2020). Reduction of liver metastasis stiffness improves response to bevacizumab in metastatic colorectal cancer. *Cancer Cell* 37 800-817.e7. 10.1016/j.ccell.2020.05.005 32516590

[B48] Sigworth F. J. (2001). Potassium channel mechanics. *Neuron* 32 555–556. 10.1016/s0896-6273(01)00509-8 11719196

[B49] Steudel F. A. Mohr C. J. Stegen B. Nguyen H. Y. Barnert A. Steinle M. (2017). SK channels modulate Ca(2+) signalling and cell cycle progression in murine breast cancer. *Mol. Oncol.* 11 1172–1188. 10.1002/1878-0261.12087 28557306 PMC5579333

[B50] Stringer B. K. Cooper A. G. Shepard S. B. (2001). Overexpression of the G-protein inwardly rectifying potassium channel (GIRK1) in primary breast carcinomas correlates with axillary lymph node metastasis. *Cancer Res.* 61 582–588. 11212253

[B51] Sun H. Luo L. Lal B. Ma X. Chen L. Hann C. L. (2016). A monoclonal antibody against KCNKK(+) channel extracellular domain inhibits tumour growth and metastasis. *Nat. Commun.* 7:10339. 10.1038/ncomms10339 26842342 PMC4742836

[B52] The L. (2018). GLOBOCAN 2018: counting the toll of cancer. *Lancet* 392:985. 10.1016/s0140-6736(18)32252-9 30264708

[B53] Trepat X. Chen Z. Jacobson K. (2012). Cell migration. *Compr. Physiol.* 2 2369–2392. 10.1002/cphy.c110012 23720251 PMC4457291

[B54] Urrego D. Movsisyan N. Ufartes R. Pardo L. A. (2016). Periodic expression of Kv10.driven by pRb/E2F contributes to G2/M progression of cancer and non-transformed cells. *Cell Cycle* 15 799–811. 10.1080/15384101.2016.1138187 27029528 PMC4845928

[B55] Wang H. Zou L. Ma K. Yu J. Wu H. Wei M. (2017). Cell-specific mechanisms of TMEM16A Ca(2+)-activated chloride channel in cancer. *Mol. Cancer* 16:152. 10.1186/s12943-017-0720-x 28893247 PMC5594453

[B56] Wang Y. Wang L. Yin C. An B. Hao Y. Wei T. (2015). Arsenic trioxide inhibits breast cancer cell growth via microRNA-328/hERG pathway in MCF-cells. *Mol. Med. Rep.* 12 1233–1238. 10.3892/mmr.2015.3558 25824027

[B57] Williams S. Bateman A. O’Kelly I. (2013). Altered expression of two-pore domain potassium (K2P) channels in cancer. *PLoS One* 8:e74589. 10.1371/journal.pone.0074589 24116006 PMC3792113

[B58] Zhang X. Zhang L. Lin B. Chai X. Li R. Liao Y. (2017). Phospholipid phosphatase promotes proliferation and tumorigenesis, and activates Ca(2+)-permeable cationic channel in lung carcinoma cells. *Mol. Cancer* 16:147. 10.1186/s12943-017-0717-5 28851360 PMC5576330

